# Reducing Bias Against Families in Low-Income Homes

**DOI:** 10.1017/jme.2025.10082

**Published:** 2025

**Authors:** Rupali Gandhi, Jill Glick

**Affiliations:** 1 Pediatric Cardiology, Advocate Children’s Hospital, Oak Lawn, United States; 2 Pediatric Cardiology, The University of Chicago, Chicago, United States; 3 Child Protective Services, Pediatrics, The University of Chicago, Chicago, United States; 4 Burn and Complex Wound Center, The University of Chicago, Chicago, United States

**Keywords:** child abuse, low income housing, child protection services, childhood burns

## Abstract

Shields and colleagues raise a concern for bias against low-income families when reporting suspected intentional scald burns. This is a plausible theory, and the development of Child Abuse Pediatrics as a specialty has likely helped reduce bias because they take the sociodemographic factors into account and are keenly aware of housing problems such as water heaters that are not regulated. Bringing their expertise to burn units will help reduce bias, and efforts should focus on public policy changes as described by the authors, but also on parental education to reduce the overall incidence of burn injuries in children.

In this issue, Shields and colleagues provided a metanalysis of 18 studies between 1970 and 2024 representing >4,000 childhood scald burns to characterize indicators of intentional scald burns in children. They found that the following characteristics were indicators for intentional scald burns: tap water as the causal agent; patterns including immersion injury, bilateral injury, and buttocks, perineum or feet injury; a child 2 years or younger; and a child of a low-income household. The authors posit that the evidence behind the “low-income household” indicator may have been subject to bias because families living in low-income homes may have an increased number of scald burns not because of higher rates of abuse, but because their homes are more likely to have hot water heaters that are either unregulated or, alternatively, regulated but still dispensing water above 120°F at the faucet.^1^ While they cannot prove the link between poorly regulated water heaters and misclassification of scald burns as intentional, the authors recommend that intentionality should be reexamined in the current clinical and criminal paradigms to consider that water may reach excessive temperatures without parental knowledge, and that sociodemographic characteristics should be analyzed more fully to minimize bias and to better understand the relationship between the burn injury and income level.

## The evolution and impact of child abuse pediatrics

Shields and colleagues argue that one way to prevent bias against those in low-income homes is to improve the child abuse investigators’ knowledge of how to assess for suspected child abuse. They argue that investigators should be given education regarding the time it takes to scald at different water temperatures and that the water temperature at the faucet may differ from the temperature set on the hot water heater. They also recommend investigators be taught to obtain photographic evidence of the set water temperature. While it is crucial to educate investigators on collecting vital data to recreate the scene of an injury, there are additional important resources that should be incorporated into the assessment of potential child abuse. In particular, child abuse medical specialists have become instrumental in the detection of child abuse. In 2006, Child Abuse Pediatrics (CAP) became a recognized pediatric subspecialty by the American Board of Pediatrics, partly in response to the demand by child welfare investigators, law enforcement, medical providers, courts and parents for medical expertise in discerning the manner of injury.^2^ To pursue this subspecialty, a physician must complete three years of general pediatrics residency, three additional years of CAP fellowship and pass the CAP board certifying exam.^3^ Child abuse pediatricians are responsible for the diagnosis and treatment of children who are suspected victims of any form of child abuse. Child abuse pediatricians use evidence-based knowledge, set standards of care for the process of discerning the manner of injury to reduce bias, improve efficiency, and refine proficiency.^4^

The efforts of child abuse pediatricians have been impactful. CAP physicians diagnose abuse or neglect less often as compared to other physicians.^5^ One single-center study of 2,495 consultations for child abuse found that although 15% of all referrals were due to burns, the CAP specialists found abuse likely in only 27% of these.^6^ Furthermore, of the total number of referrals for suspected abuse, CAP consultants found definite or likely abuse in only 40% of cases.^7^ This likely reflects the referring providers’ uncertainty in assessing the etiology of injury and the ability of the CAP physician to discern the manner of injury due to their expertise.

In 2017, Collier and colleagues classified the manner of injury of children admitted to the University of Chicago Burn Unit, a center where all children are assessed by a child abuse pediatric team, social workers, the burn unit team, and investigators to reach consensus regarding manner of injury. Assessment included a meticulous history of presentation, scene reenactment at home by authorities and psychosocial evaluation. Over a 6-year period, 408 children were admitted, and the average age was 2.9 years; 8% of burns were deemed due to abuse, 7% due to neglect, and 85% accidental.^8^ With a far lower rate of abuse-related burns compared to the current study, it is likely that with a comprehensive multidisciplinary assessment and appropriate expertise, more burn injuries would be deemed accidental.

## Prevention of accidental scald burns

Since the overwhelming number of burns in children are deemed unintentional by child abuse experts, focusing on injury prevention can have a dramatic impact on reducing these accidents.

Shields and colleagues argue that changes in building codes and requiring mixing valves with water heater replacements would decrease the incidence of scald burns. They also contend that rental assistance programs that require home safety inspections should include tap water temperatures in their assessments. Legislative efforts such as these are important but can take significant time to be enacted.

A more immediate intervention is to focus on parental education. Pediatricians routinely counsel on safety and accident prevention as a part of every well-child visit because accidental injuries are so common in childhood. Parents are more likely to listen to their pediatrician with whom they have built a trusting relationship, and the counseling can be repetitive with age-specific advice as the child grows. The American Academy of Pediatrics *Bright Futures* initiative provides evidence-based guidelines for what pediatricians should discuss at each visit.^9^ There are specific guidelines to discuss safe bathing practices with parents including keeping water < 120°F, adjusting the water heater if needed, never leaving the baby alone in water, and checking the temperature with one’s wrist to ensure it is not too hot.^10^ Pediatricians are also in a good position to give updated advice to parents when newer data is released such as the finding that 95% of scald burns over a ten-year period at a single center were associated with running water, and that recommending that parents turn off the water after filling the tub will likely significantly decrease accidental burns.^11^

In conclusion, Shields and colleagues raise a concern for bias against low-income families when reporting suspected intentional scald burns. This is a plausible theory, and the development of Child Abuse Pediatrics as a specialty has likely helped reduce bias because they take the sociodemographic factors into account and are keenly aware of housing problems such as water heaters that are not regulated. Unfortunately, there are only 389 board-certified Child Abuse Pediatricians as of 2024. There are many hospitals and several states without a single Child Abuse Pediatrician.^12^ Therefore, it remains crucial to encourage the growth of CAP as a specialty and strongly advise that all Burn Centers have a CAP consultant accessible. Bringing their expertise to burn units will help reduce bias, and efforts should focus on public policy changes as described by the authors, but also on parental education to reduce the overall incidence of burn injuries in children.

## References

[1] W.C. Shields, et al., “Characteristics of Scald Burns Caused by Child Abuse in the United States: A Systematic Review,” Journal of Law, Medicine & Ethics 53 no. 2 (2025): 230–241. 10.1017/jme.2025.10111. 40621666

[2] R.W. Block and V. J. Palusci, “Child Abuse Pediatrics: A New Pediatric Subspecialty,” Journal of Pediatrics 148 no. 6 (2006): 711–712. 16769370 10.1016/j.jpeds.2006.01.033

[3] “Program Requirements, FAQs, and Applications,” Accreditation Council for Graduate Medical Education, https://www.acgme.org/specialties/pediatrics/program-requirements-and-faqs-and-applications/ (last visited March 5, 2025).

[4] S. Kleinle et al., “A History of Child Abuse Pediatrics: Training, Research, and Clinical Diagnosis,” Rhode Island Medical Journal 106 (November 2023):10–14, at 12. 37890057

[5] Id.

[6] R.A. Hicks et al., “Consultations in Child Abuse Pediatrics,” Clinical Pediatrics 59, no.8 (2020): 809–815. 32418448 10.1177/0009922820920019

[7] Id.

[8] Z.J. Collier et al., “A 6-Year Case-Control Study of the Presentation and Clinical Sequelae for Noninflicted, Negligent, and Inflicted Pediatric Burns,” Journal of Burn Care & Research 38, no. 1 (2017): e101–e124. 28009699 10.1097/BCR.0000000000000408

[9] “Bright Futures Guidelines and Pocket Guide,” American Academy of Pediatrics, https://www.aap.org/en/practice-management/bright-futures/bright-futures-materials-and-tools/bright-futures-guidelines-and-pocket-guide/?srsltid=AfmBOoqUoZXN2abbtfKp00DYmqhzJVWKa1f-5phDIQm5tak7cknhmqpz (last visited March 5, 2025).

[10] Id.

[11] W.J. Moser et al., “Running Water While Bathing is a Risk Factor for Pediatric Scald Burns,” Burns 49, no. 7 (2023): 1714–1718 37193613 10.1016/j.burns.2023.03.014

[12] “Pediatric Subspecialists Ever Certified,” The American Board of Pediatrics, https://www.abp.org/dashboards/pediatric-subspecialists-ever-certified (last visited March 5, 2025).

